# Exploring Mechanisms and Biomarkers of Breast Cancer Invasion and Migration: An Explainable Gene–Pathway–Compounds Neural Network

**DOI:** 10.1002/cam4.70769

**Published:** 2025-03-17

**Authors:** Xia Qian, Dandan Sun, Yichen Ma, Ling Qiu, Jie Wu

**Affiliations:** ^1^ Department of Laboratory Medicine Peking Union Medical College Hospital, Peking Union Medical College & Chinese Academy of Medical Science Beijing People's Republic of China; ^2^ State Key Laboratory of Complex Severe and Rare Diseases Peking Union Medical College Hospital, Peking Union Medical College & Chinese Academy of Medical Science Beijing People's Republic of China

**Keywords:** breast cancer, cancer genomics, interpretable neural networks

## Abstract

**Backgrounds:**

Exploring the molecular features that drive breast cancer invasion and migration remains an important biological and clinical challenge. In recent years, the use of interpretable machine learning models has enhanced our understanding of the underlying mechanisms of disease progression.

**Methods:**

In this study, we present a novel gene–pathway–compound‐related sparse deep neural network (GPC‐Net) for investigating breast cancer invasion and migration. The GPC‐Net is an interpretable neural network model that utilizes molecular data to predict cancer status. It visually represents genes, pathways, and associated compounds involved in these pathways.

**Results:**

Compared with other modeling methods, GPC‐Net demonstrates superior performance. Our research identifies key genes, such as ADCY8, associated with invasive breast cancer and verifies their expression in breast cancer cells. In addition, we conducted a preliminary exploration of several pathways.

**Conclusion:**

GPC‐Net is among the pioneering deep neural networks that incorporate pathways and compounds, aiming to balance interpretability and performance. It is expected to offer a more convenient approach for future biomedical research.

## 1 Introduction

Traditional machine learning methods, such as logistic regression and decision trees, are known for their high interpretability but often have lower prediction performance. In contrast, deep learning has shown superior performance in various applications but lacks interpretability due to the “black box effect” of neural networks. However, when exploring disease mechanisms or molecular expression functions, models lacking interpretability are often unsatisfactory. This is especially true when attempting to identify new mechanisms or biomarkers, as interpretable models offer more advantage [1, 2, 3]. Therefore, when developing new predictive models, it is important to strike a balance between interpretability and high performance. Currently, there are two widely adopted strategies for creating interpretable neural networks. The first is to interfere or calculate the marginal contribution to a single input or neuron and observe its effect on subsequent neurons in the neural network, exemplified by ZFNet [4], LIME [5], SHapley Additive exPlanations [6, 7] and GNNExplainer [8]. Alternatively, neural networks such as DeepLIFT [9], Integral Gradient [10], and LRP [11] can analyze the contribution of each level of the input layer by backpropagating important signals from the output neuron through each layer to the input neuron. Recently, Eliezer M. Van Allen et al. proposed a sparse connection model, that explains the layer‐by‐layer transfer of neural networks based on the relationship between genes and pathways [12]. This model exhibits exceptional performance while demanding fewer computational resources. By comparing the weighted link relationship between genes and pathways, we can not only identify the functioning genes but also understand how they work. This groundbreaking study has somewhat opened up the “black box” of biomolecular‐related bioinformatics analysis.

With the advancements in molecular biological detection techniques, it is now possible to clearly present the gene expression, epigenetic signatures, and other molecular profiles of cancer patients [13, 14]. The role of these molecular characteristics in disease progression, particularly in the case of tumors, is a topic of ongoing research and discussion. Numerous biological pathways and processes have been verified to play a crucial role in disease development, targeted drug discovery, drug resistance, and prognosis [15, 16, 17]. At present, most of the research on pathways aims to identify biomarkers within these pathways for diagnosing or predicting disease progression, as well as identifying potential target substances to inhibit or enhance their effects for therapeutic purposes [18, 19]. In biological pathways, there are numerous compounds (a collection of small molecules, biopolymers and other chemicals associated with biological systems), which serve as the embodiment of the pathway. Identifying crucial compounds within these pathways is a current research focus, aiming to explore disease mechanisms or establish them as targets for therapy or diagnosis. While existing models leverage biological prior knowledge to improve interpretability, they often focus either on gene–pathway relationships or compound‐level interactions separately. GPC‐Net presents a novel approach by integrating gene, pathway, and compound levels into a single sparse hierarchical neural network, thereby enhancing interpretability without sacrificing predictive power. To facilitate the identification of these potential pathways and biomarkers, and to gain a more intuitive understanding of disease mechanisms, we have developed a gene–pathway–compound neural network (GPC‐NET) using sparse links. In the construction of GPC‐NET, we carefully curated 352 pathways, including metabolic pathways and signaling pathways, to serve as bridges. Additionally, we extracted 3371 compounds, including metabolites within these pathways, to construct a pathway‐ and compound‐aware multilayered hierarchical deep network. In GPC‐NET, the patients' entire gene profile, encompassing gene mutation, amplification, deletion, and methylation features, is inputted into the neural network. The genes are then distributed across the nodes of the first layer through weighted links. The second layer of the network encodes the curated biological pathways, while the third layer represents the various compounds present in these pathways. Sparse links are established between different layers based on known and recognized child–parent relationships, ensuring the interpretability of the neural networks.

## 2 Result

### 2.1 GPC‐Net

**FIGURE 1:**
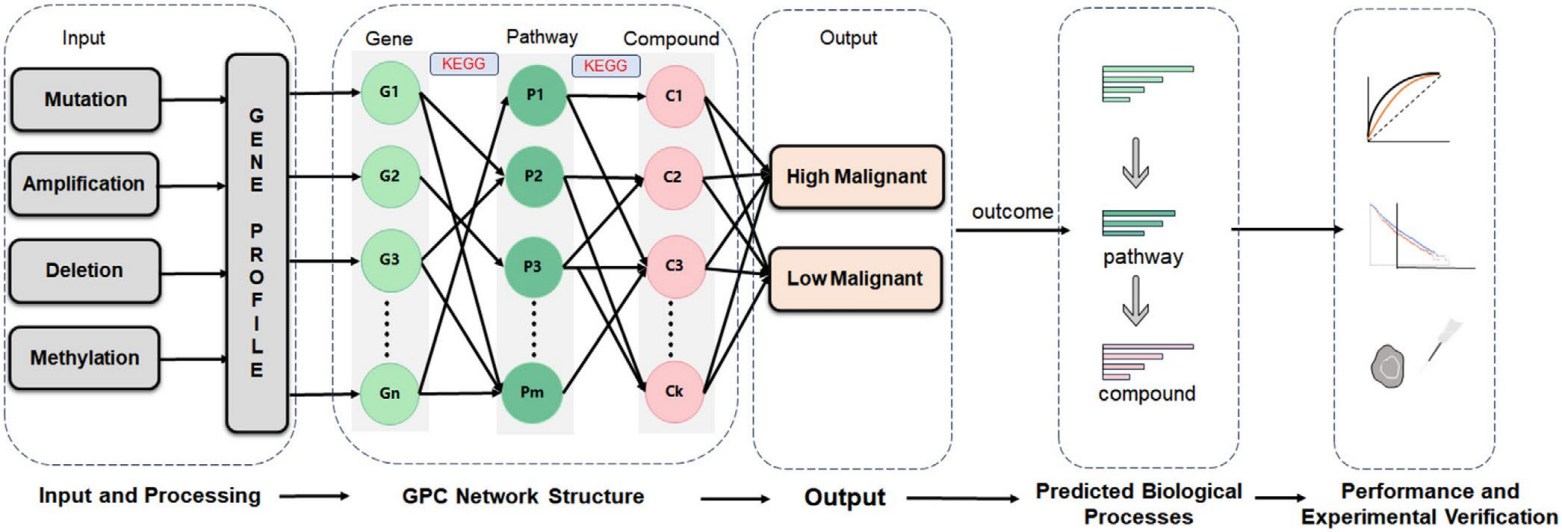
Workflow of GPC‐NET. GPC‐NET comprises upstream and downstream relationship connections of genes, pathways, and compounds to form a sparse deep neural network. The patient's whole genome information is inputted, and the pathological grading of breast cancer cells is used as the output label to predict the malignancy of cancer cells. The predefined connections between the gene, pathway, and compound layers based on KEGG database annotations enable the interpretation of the cancer mechanism through a gene–pathway–compound activation connection.

GPC‐NET is an interpretable neural network that encodes the superior–subordinate connections among genes, pathways, and compounds as neural network connections. In this study, we trained GPC‐NET using gene profiles of breast cancer patients, including gene mutation, amplification, deletion, and methylation (Figure 1). Each node in each layer is assigned a weight score, and important genes, pathways, and compounds are ranked based on this score. The top factors were then validated to explore their further function. To train and test GPC‐NET, we utilized a set of 1892 breast cancer samples, divided into 85% for training, 5% for validation, and 10% for testing, to investigate the mechanisms underlying tumor cell invasion and migration enhancement. The complete gene profile of each patient was input into GPC‐NET.

In terms of tumor differentiation, poorly differentiated tumors are characterized by high malignancy, rapid growth, a high metastasis rate, and a poor prognosis. Conversely, well‐differentiated tumors exhibit low malignancy, slow growth, a low metastasis rate, and a favorable prognosis [20]. Thus, to investigate the mechanisms associated with the degree of invasion, metastasis, and malignancy of breast cancer cells, we hypothesized that poorly differentiated and undifferentiated cells represent highly malignant cancer cells (HMC, 952 samples), while the opposite is true for low malignant cancer cells (LMC, 940 samples).

### 2.2 Prediction Performance of GPC‐NET

**FIGURE 2:**
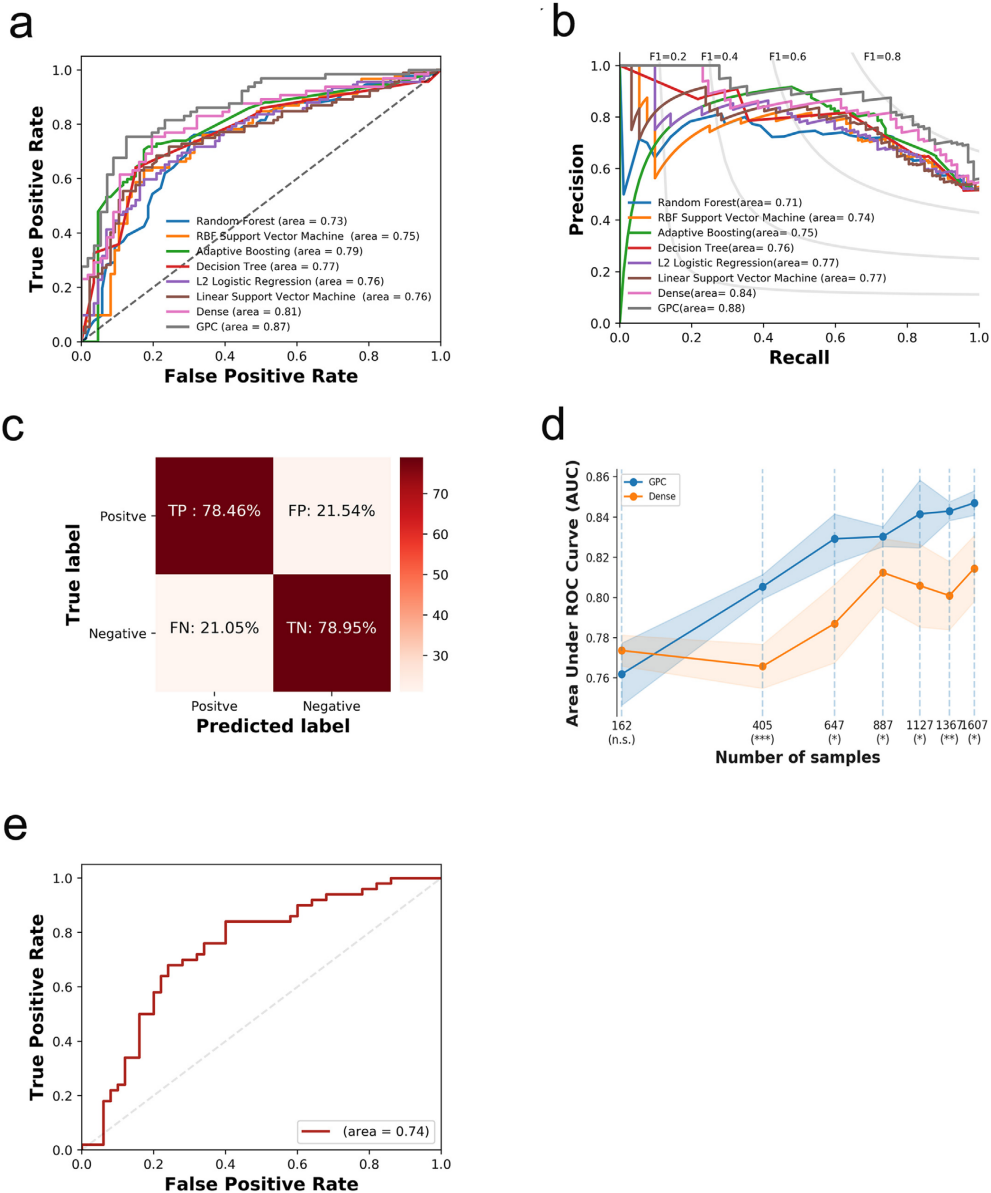
Prediction performance of GPC‐NET. In terms of the test set performance, the prediction performance of GPC‐NET is better than that of other models. (a) Area under the receiver operating characteristic (ROC) curve (AUC), values shown in brackets. (b) Area under the precision‐recall curve (AUPRC), values shown in brackets. (c) GPC‐NET achieves a 78.46% true‐positive rate (TP) and a 78.95% true‐negative rate (TN), FN, false‐negative rate; FP, false‐positive rate. (d) In different sample sizes, the prediction performance (AUC) of GPC‐NET was better than that of the dense fully connected deep neural network model. (*p < 0.05, **p < 0.01; ***p < 0.001). (e) An independent external validation cohort was used to evaluate GPC‐NET.

**FIGURE 3:**
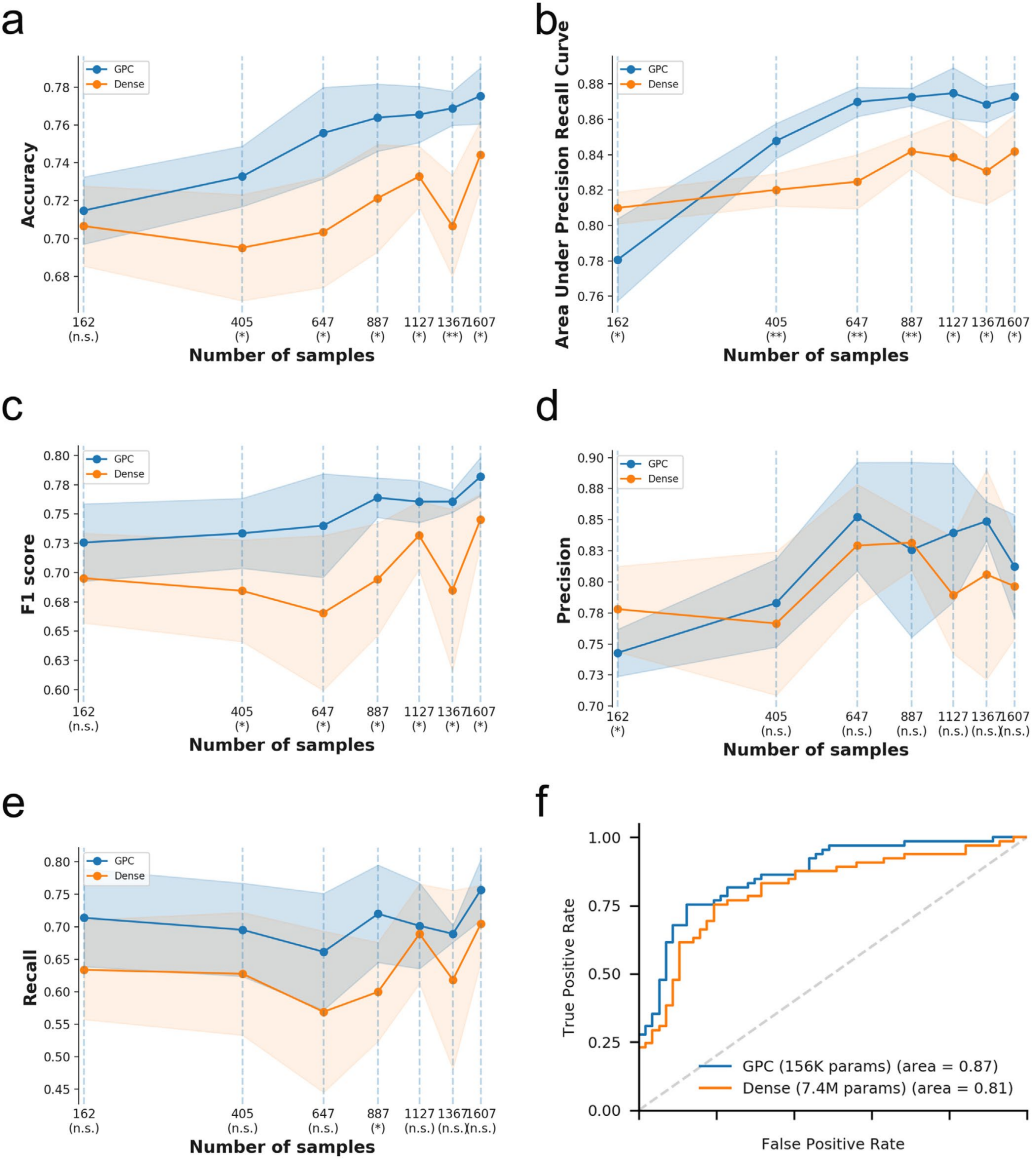
Comparison of GPC‐NET to dense models. The comparative analysis involved evaluating the prediction performance of a dense fully connected neural network and GPC‐NET across varying sample sizes. (a: Accuracy, b: AUPRC, c: F1 score, d: Precision, e: Recall, *p < 0.05, **p < 0.01, the solid line in the graph depicts the average value, while the bands surrounding it represent the average value +/− SD. This data is based on a five‐fold cross‐validation experiment, f: Comparison of AUC and performance parameters between GPC‐NET and dense models).

The predicted performance of the trained GPC‐NET is superior to that of traditional machine learning models such as random forest, support vector machine, logistic regression, and decision tree. This is evident in metrics such as area under the receiver operating characteristic (ROC) curve (AUC) of 0.87, accuracy of 0.79, area under the precision–recall curve (AUPRC) of 0.88, precision of 0.81, recall of 0.78, and f1 score of 0.80 (Figure 2). Furthermore, when comparing GPC‐NET to a fully connected dense neural network, GPC‐NET outperformed the dense model. We also conducted a comparison of the two models using different training sample sizes and found that GPC‐NET consistently outperformed the dense neural network in terms of predictive performance (AUC) (Figure 2d). Additionally, GPC‐NET performed better than the dense fully connected model in other prediction performance metrics, such as accuracy, AUPRC, F1 score, precision, and recall (Figure 3a–e). GPC‐NET consistently outperformed the dense model while using fewer parameters (Figure 3f). To validate the performance of GPC‐NET, we used the MBC project dataset as an independent external validation, which included 89 high‐risk metastatic cancer (HMC) samples and 111 low‐risk metastatic cancer (LMC) samples. Despite lacking methylation data, this cohort still achieved an AUC of 0.74, indicating that GPC‐NET demonstrates generalization capabilities with unseen datasets (Figure 2e).

### 2.3 Analysis of the Gene Layer

**FIGURE 4:**
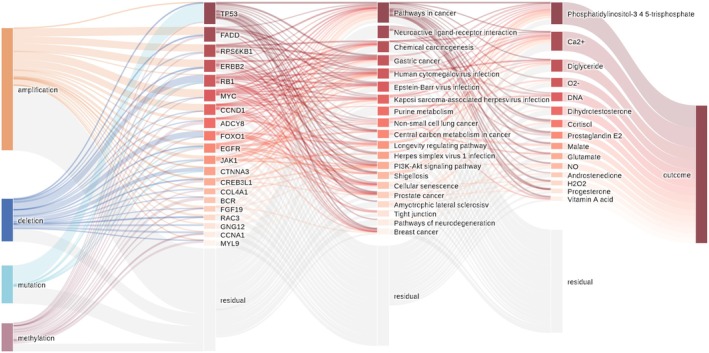
Visualization of GPC‐NET. The visual representation of GPC‐NET demonstrates the interpretability of the analysis. The input features (amplification, deletion, mutation, and methylation) are depicted on the leftmost side, connected through the gene layer, pathway layer, and compound layer, ultimately leading to the output result. The color depth and line width represent the importance and contribution of each component. For instance, TP53 is primarily characterized by mutations, while FADD is mainly characterized by amplification.

To provide a clearer explanation of GPC‐NET after training, we visualized the inner layers and stream of the neural network (Figure 4). We found that copy number variation, particularly amplification, contributed the most to DNA molecular changes, which is consistent with previous reports [21]. The importance of genes in the first layer was assessed, and the top 20 genes were selected based on their rank. This selection included genes likeTP53, FADD, RPS6KB1, and ERBB2 (Figure S1). Molecular alterations, including gene amplification, deletion, mutation, and methylation, were further explored. Among these alterations, gene amplification had the most significant impact, followed by gene deletion and mutation.

**FIGURE 5:**
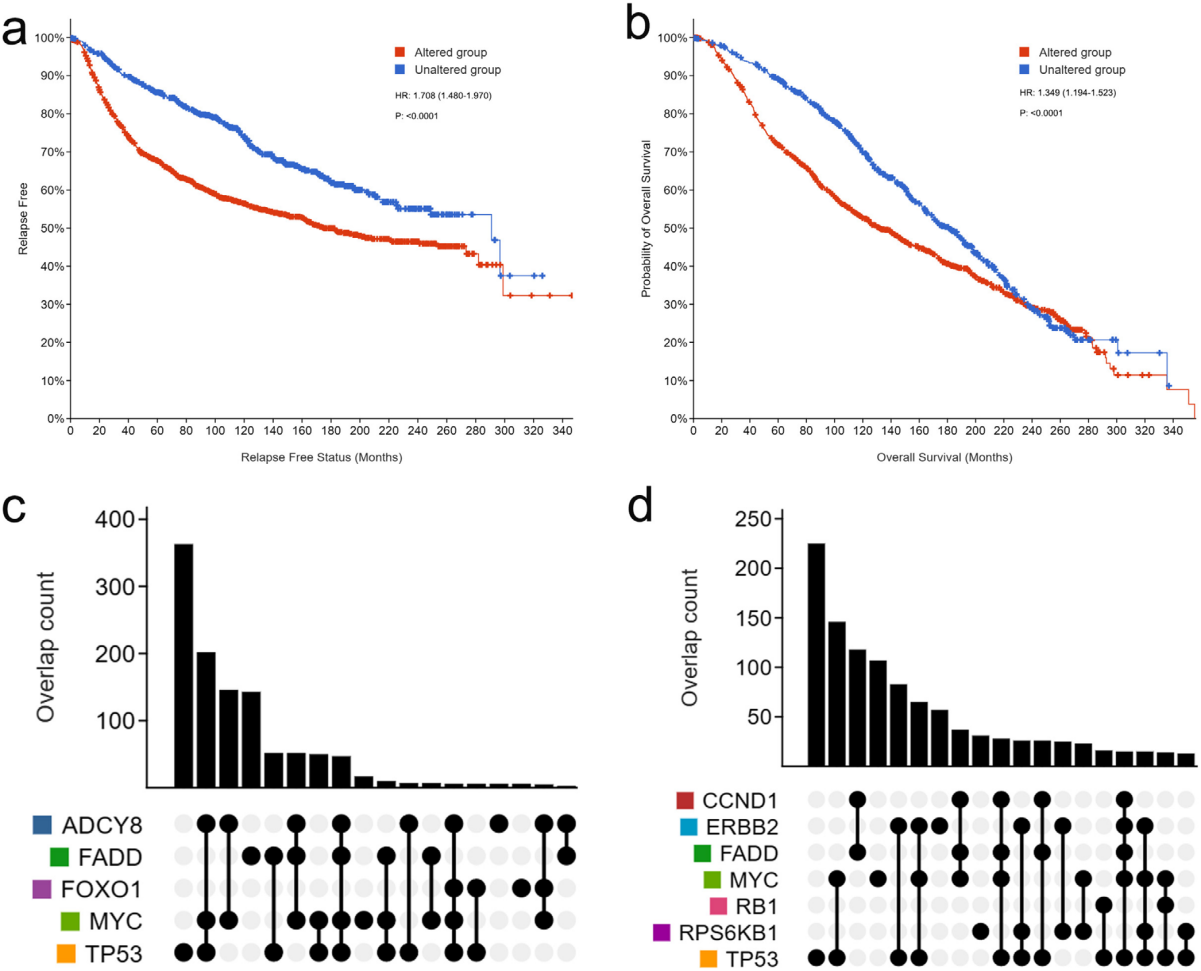
Analysis of the gene layer. (a) Relapse‐free status curve of patients showed the time period without locoregional relapse, distant relapse, or death‐specific death. The TOP gene altered group had a median of 182.43 months (range 160.23–240.95), while the TOP gene unaltered group had a median of 290.89 months (range 226.25—NA) (*p* < 0.00001). (b) The overall patient survival status curve (TOP gene altered group, 132.03 (119.37–147.77); TOP gene unaltered group, 181.23 (165.40–197.33), *p* < 0.00001). (c) Co‐alteration of ADCY8 with MYC and TP53. (d) Co‐alteration of some TOP genes.

To investigate the role of the top genes, those with mutations, deep deletions, or significant amplification were classified as an altered group, while the group without the above changes in top gene was defined as the unchanged group. The relapse‐free survival status and overall survival status of patients in the altered group was significantly lower than that in the unaltered group, indicating that when these top genes were molecularly altered, metastasis was more likely to occur, resulting in a poorer prognosis compared to the unaltered group (Figure 5a,b). Furthermore, among the top genes, TP53 played the most important role, particularly in terms of mutations.

**FIGURE 6:**
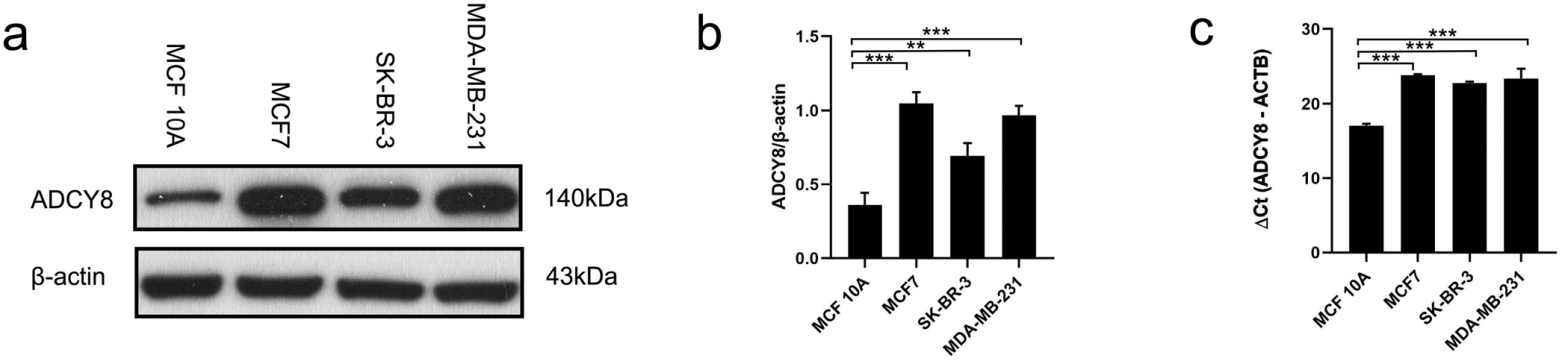
RNA and protein expression of ADCY8. (a) Immunoblotting was conducted on human normal breast cells, MCF 10A, and different breast cancer cell lines, including MCF7, SK‐BR‐3, and MDA‐MB‐231. (b) Quantitative analysis of the immunoblotting results. (c) The expression of ADCY8 mRNA in different cells was detected by Real‐time quantitative PCR. LSD (Least Significant Difference), **p < 0.01, ***p < 0.001.

Among these genes, in addition to genes that are clearly known to influence breast cancer proliferation or prognosis, we identified a gene, ADCY8, that has been relatively poorly studied in breast cancer, showing overlapping alterations with TP53 and MYC (Figure 5c,d). The protein expression of ADCY8 was found to be higher in breast cancer tissues compared to normal breast tissues, suggesting its potential role in breast cancer progression (Figure S2). This finding was further supported by the mRNA and protein expression analysis in multiple breast cancer cell lines (Figure 6). Breast cancer cells and breast adenocarcinoma cells exhibited higher ADCY8 expression compared to normal breast cells, indicating a specific role for ADCY8 in breast cancer cells.

### 2.4 Analysis of the Pathway Layer and Compound Layer

In the layer of the pathway analysis, it was observed that many of the highest‐ranked pathways were common pathways involved in epithelial tumor formation and oncogenesis. This suggests that regardless of the specific type of epithelial tumor, the mechanisms governing their proliferation, invasion, and metastasis may be similar. To gain further insight into the specific roles of these pathways, the results of GPC‐NET analysis were categorized and summarized using KEGG's pathway database (Table S2). We found that, in addition to these common global and overview maps pathways in epithelial tumors, the most important metabolism is purine metabolism (Figure 4). In order to better understand its role in breast cancer and verify the accuracy of GPC‐NET, we further explored it. Previous studies have shown that purine metabolism regulates the malignant behavior of various cancers and their response to immune checkpoint inhibitors [22, 23]. Genes involved in purine metabolism were extracted from the KEGG database and it was observed that mutations, amplifications, and deep deletions in purine metabolism genes were associated with increased metastasis and decreased survival (Figures S3a, S3b). In addition, we further explored the tyrosine, which is most tightly linked to the Top gene. But it is worth noting that the role of tyrosine metabolism in breast cancer is not frequently mentioned. To explore this further, urine samples were collected from 79 patients with breast tumors, including 51 with invasive cancer and 28 with noninvasive cancer (carcinomas in situ or breast fibroadenomas), based on surgical pathology biopsy. The results showed that patients with invasive cancer had higher levels of tyrosine excretion compared to those with benign tumors and carcinoma in situ. We further analyzed the genes involved in tyrosine metabolism and found that patients with altered tyrosine metabolism had shorter survival times and were more likely to develop metastases, indicating a close association between tyrosine metabolism and breast cancer cell invasiveness.

## 3 Discussion

The GPC‐NET is a structured sparse model constructed using bioinformatics networks. The study highlights the superior prediction performance of GPC‐NET compared to traditional machine learning models such as random forest, support vector machine, logistic regression, and decision tree. Notably, GPC‐NET outshines a fully connected dense neural network in various performance metrics while utilizing fewer parameters, a testament to its efficacy in capturing nuanced relationships within the dataset. Additionally, the entire process is visually represented, allowing for direct observation of not only the genes and pathways involved in tumor metastasis but also the effects of compounds within each pathway. This visual insight facilitates in‐depth exploration into the underlying mechanisms of tumor metastasis, providing a wealth of information for identifying novel therapeutic targets tailored to molecularly stratified populations. By constructing mechanistic prediction models, GPC‐NET presents a promising approach to integrating cancer biology with machine learning, providing a versatile platform applicable to mechanistic investigations in cancer research.

Figure 4 clearly shows the role of each gene in the progression of breast cancer; that is, the first‐layer genes play functions through the second‐layer pathways and third‐layer compounds, which help us to more intuitively observe important biomarkers and provide new biomarker ideas for diagnosis and treatment. Genes can be considered one of the fundamental causes underlying changes in disease. Many of the top genes identified in GPC‐NET have well‐established functions in cell cycle regulation, invasion, and metastasis of breast cancer cells. These genes, including TP53 [24], FADD [25, 26], RPS6KB1 [27], ERBB2 [28], RB1 [29], MYC [30, 31], CCND1 [32], and EGFR [33], have been linked to poor prognosis in cancer. We have uncovered a novel gene, ADCY8, which has been largely overlooked in studies concerning the invasion and metastasis of breast cancer. Notably, this gene demonstrates a high likelihood of concurrent alterations with TP53 and MYC (Figure 5c,d). The expression and alteration patterns of TP53 and MYC, along with their associated genes, often serve as indicators of changes in prognosis [34]. Intriguingly, prior research has highlighted the significance of TP53 and ADCY8 in somatic mutations associated with early lung squamous cell carcinoma [35]. Moreover, gender‐specific implications of ADCY8 have been revealed, portraying it as a risk factor in female glioma patients while exhibiting a protective role in male patients [36]. This gender‐specific nuance further underscores the substantial role that ADCY8 may play in breast cancer, a disease with a notable female predisposition. In this study, ADCY8 emerged as a significant key gene. We found a significant increase in the mRNA and protein expression of ADCY8 in breast cancer cells compared with normal breast cells, and the expression of ADCY8 is primarily located in the cytoplasmic/membranous region (Figure S2). While GPC‐Net successfully identified ADCY8 as a potential biomarker for breast cancer invasion and migration, its specific role in breast cancer metastasis remains to be further validated. Future studies should aim to perform functional experiments, such as Transwell invasion assays, siRNA‐mediated knockdown, overexpression studies, and in vivo metastasis models, to establish a direct mechanistic link between ADCY8 and tumor progression. Additionally, investigating downstream signaling pathways and interacting partners of ADCY8 in breast cancer cells will provide deeper insights into its biological function.

Metabolism pathway is an indispensable biological process that is the basis of all life activities. Because of their special moldable and adaptable metabolism, tumor cells possess a character that can make them unlimitedly grow and divide even in a harsh environment of nutritional deficiency. Adaptive enhancement of tumor cell metabolism allows them to fight for more energy to survive than normal cells. Ever since Warburg discovered that tumor cells metabolize by glycolysis rather than aerobic respiration, exploring how the metabolism of tumor cells changes has always been one of the keys to studying the growth mechanism of cancer [37]. Recently, a study has found that the energy metabolism of solid tumor cells seems to be more frugal, which overturns the previous view that tumor cells occupy huge energy [38]. Therefore, finding the metabolism or pathway differences of cancer cells is still a problem that we need to pay attention to [39]. Furthermore, the ranking of the three main metabolisms in terms of their weight was examined (Table S2). In carbohydrate metabolism, amino acid and nucleotide metabolism play an important role, which is consistent with the increased demand for nucleotides and amino acids during cancer cell proliferation and metastasis. Glycolysis/gluconeogenesis and the citrate cycle (TCA cycle) closely followed, indicating that aggressive metastasis in cancer cells requires significant energy. In lipid metabolism, steroid hormone biosynthesis was found to be the most important, with metabolites related to the steroid hormone pathway, such as dihydrotestosterone and progesterone, also ranking highly. This is consistent with the known role of steroid hormones, such as estrogen and progestins, in breast cancer development [40]. Additionally, phosphatidylinositol‐3,4,5‐trisphosphate and Diacylglycerol were among the top‐ranked metabolites in the compound layer. In terms of amino acid metabolism, cysteine, methionine, glutathione, tryptophan, and tyrosine metabolism were found to contribute the most. Some of them have been previously implicated in cancer development and prognosis [41, 42, 43]. In the hierarchical structure of KEGG gene pathways, purine metabolism is closely positioned at the forefront of metabolic processes. The pivotal role of purine metabolism has been established in other types of cancer [44, 45]. In breast cancer, impaired purine metabolism was found to increase the likelihood of metastasis and shorten survival time (Figures S3a, S3b). This further underscores the crucial role of purine metabolism in breast cancer, and its specific mechanism merits further investigation in future experiments. Furthermore, we have also observed that tyrosine metabolism appears to influence patients' survival time and cancer cell metastasis to some extent. An increased level of tyrosine metabolism was noted in patients with invasive breast cancer. Although we have made preliminary efforts to investigate tyrosine metabolism in amino acid metabolism, there are considerable limitations (Figures S3c, S3d). The overall human metabolism is intricate and susceptible to various interferences, making it challenging to demonstrate changes in cellular metabolism within the human body (through urine metabolic detection). Therefore, further experiments are needed to explore the specific mechanisms.

While GPC‐Net aimed to provide enhanced interpretability by incorporating predefined connections based on KEGG database annotations, its interpretability and performance still need improvement. The interpretability of GPC‐Net also requires further verification. For instance, the key gene ADCY8 identified by GPC‐Net should be validated using other methods such as machine learning combined with the SHAP feature selection strategy. This comparison would help determine whether GPC‐Net offers additional insights or simply replicates findings that could be obtained through conventional machine learning approaches. The limitation of our study is that for ADCY8, we only conducted RNA expression and protein expression difference validations. Further comprehensive functional studies are necessary to fully understand the role of ADCY8 in breast cancer progression. Specifically, in vivo and in vitro experiments, including invasion and metastasis assays, should be performed. For instance, using a Matrigel‐coated Transwell system to evaluate the ability of breast cancer cells to invade through the extracellular matrix. Additional experiments will help elucidate the functional role of ADCY8 in breast cancer and its potential as a therapeutic target.

The KEGG database is one of the most comprehensive and widely used resources for understanding gene–pathway relationships across various diseases and species. It integrates curated knowledge from published studies and experimental data, providing a solid foundation for pathway‐based analyses. Some genes and pathways included in KEGG may have been initially discovered in other diseases, and their specific roles in breast cancer may not yet be fully validated. A key advantage of GPC‐Net is its ability to explore these potential gene–pathway interactions that have not been previously characterized in breast cancer. By leveraging multiomics data, GPC‐Net can identify novel gene–pathway relationships that were initially observed in other diseases, thereby uncovering new biomarkers and therapeutic targets. Future enhancements to GPC‐Net could incorporate additional pathway resources such as Reactome, BioCarta, and Pathway Commons to ensure broader coverage. Moreover, future improvements should aim to integrate a more diverse range of databases and resources. For instance, incorporating drug target databases could help refine treatment strategy development. Additionally, further exploration of the model's interpretability and practical utility in biomedical research may provide a more comprehensive and reliable tool for cancer research and drug discovery.

In addition, since GPC‐Net is trained on publicly available datasets, misclassification issues may arise due to inherent data biases, a common challenge in large‐scale machine learning studies. While our model improves interpretability by mapping gene–pathway–compound relationships, further validation on independent clinical datasets is essential to enhance robustness and minimize risks. Future work will focus on external validation in diverse populations and integrating uncertainty estimation to improve model reliability for potential clinical applications.

## 4 Methods

### 4.1 GPC‐NET, Train and Optimization, Evaluation and Statistical Analysis, Selection of Important Neurons

We present the deep neural network method in detail, including the architecture design, training and optimization, evaluation and statistical analysis, and selection of important neurons.

### 4.2 Architecture Design

We introduce GPC‐NET, a neural network that reliably predicts the malignancy of tumor cells based on the genetic profiles of breast cancer patients. Illustrated in Figure 1, the comprehensive GPC‐NET model comprises five layers: the input layer, three hidden layers, and the output layer. These hidden layers crucially represent genes, pathways, and compounds, forming the fundamental core of GPC‐Net.

Within the input layer, we extend our approach by incorporating not only the three conventional gene features: mutation, amplification, and deletion, but also introducing methylation gene features. These diverse genetic attributes are then effectively channeled into the gene hidden layer through a dense net, represented as:

$$h_{i}^{1}=f\left(\sum_{j=0}^{3}W_{\mathrm{ij}}^{0}*x_{\mathrm{ij}}+b\right)$$

where $h_{i}^{1}$ is the ith gene value in gene hidden layer, $x_{\mathrm{ij}}$ is the *j*th feature of *i*th gene and $W_{\mathrm{ij}}^{0}$ represents the corresponding weight, *b* is bias value, and *f* is the tanh activation function. In the hidden layers, each node in the first hidden layer of GPC‐NET represents a gene, each node in the second hidden layer represents a pathway, and each node in the third hidden layer represents a compound, constituting a sparse linkage through its known upstream and downstream relationships in KEGG database (https://www.kegg.jp/kegg/). Parent–child connections of nodes in each layer by multiplying a mask matrix *M* by the weight matrix *W* and removing nonexisting connections according to the KEGG database. Each layer's output is determined using the formula (*f* is the activation function, *M* is the mask matrix, *W* is the weights matrix, *x* is the input matrix, *b* is the bias vector, and * is the Hadamard product):

$$H^{i}=f\left[{\left(M^{i}*W^{i}\right)}^{T}x^{i}+b^{i}\right]\;i\in \left[1,2,3\right]$$

The activation of each node was kept in the range [−1, 1] weighted input by the tanh function:

$$f=\tanh\left(x\right)=\frac{e^{x}-e^{-x}}{e^{x}+e^{-x}}$$

Finally, the output layer outputs the grade score of the breast cancer using the sigmoid function:

$$S\left(x\right)=\frac{1}{1+e^{-x}}$$

the grade score is in the range [0, 1], where 0 denotes well‐differentiated and moderately differentiated, and 1 denotes poorly differentiated and undifferentiated.

As the node connections are established using relationships in the KEGG database, our model achieves enhanced interpretability and prediction performance with minimal parameters. Notably, in our model, the hidden layers consist of 18,611, 352, and 3378 nodes for genes, pathways, and compounds, respectively. The total parameter count in our sparse network is 7.4 K, representing a remarkable reduction of 0.047 times compared with the 156 K parameters in the fully connected dense network.

### 4.3 Utilizing KEGG Annotations

Gene–pathway mapping: Each gene in the gene layer is connected to its associated pathways according to KEGG database annotations. This mapping ensures that gene alterations can influence their related pathways.

Pathway–compound mapping: Pathways identified in the KEGG database are connected to compounds that are involved in these pathways. This hierarchical connection allows the network to trace the impact of gene alterations through pathways down to the compound level.

Predefined connections: The connections between genes, pathways, and compounds are predefined based on the KEGG database to ensure interpretability. This structured approach allows for the identification of gene–pathway–compound activation connections, which helps in interpreting the mechanisms underlying cancer.

### 4.4 Train and Optimization

We trained GPC‐NET using genomic data from 1892 patients (952 HMC and 940 LMC), including mutations, amplifications, deletions, and methylations (data, “Aparicio S et al.” cohort [46, 47, 48]).

The model was trained using the binary cross‐entropy loss function:

$$H=-\frac{1}{N}\sum_{i=1}^{N}y_{i}\log\left(p\left(x_{i}\right)\right)+\left(1-y_{i}\right)\log\left(1-p\left(x_{i}\right)\right)$$

where *y*_i_ is the label for sample *x*_*i*_, *p* (*x*_*i*_) is the predicted grade score, and *N* is the total number of samples. To allow each layer to be useful by itself, we added a predictive layer with sigmoid activation after each hidden layer. Therefore, the overall objective function of the model is composed of *three loss functions*, *as follows*:

$$H_{\text{total}}=\alpha_{1}*H_{1}+\alpha_{2}*H_{2}+\alpha_{3}*H_{3}$$

where $\alpha_{i}$ denotes the weight of the corresponding loss.

Table S1 shows all values of hyperparameters in our models. The optimizer is Adam, the learning rate was initialized to be 0.008 and actively reduced after every 50 epochs to allow for smooth convergence. Empirically, we found that using an adaptive learning rate besides Adam led to smoother convergence and improved prediction performance.

### 4.5 Evaluation and Statistical Analysis

The prediction performance was measured using the average AUC, the AUPRC, the accuracy, and the F1 score. The corresponding measures were reported for the train‐validation‐testing split and also for the crossvalidation setup. For the train‐validation‐testing split set, the input data were split randomly into three sets for training, validation, and testing, with a ratio of 0.85:0.05:0.1. For the crossvalidation experiments, the dataset was divided into five folds stratified by the label classes to account for the bias in the dataset.

To verify the generalization ability of the model, we introduce an external dataset for evaluation. The external test results are produced by a model that is trained on the main dataset and tested on one independent external dataset. The implementation of the proposed system, along with the reproducible results, is available on GitHub (https://github.com/286331722/Gene_Path_Compound_Neural_Network).

The change in the area under the ROC curve between GPC‐net and other models is tested using the DeLong test [49]. The *p* values have been adjusted for multiple hypothesis testing using the false discovery rate (FDR). To address multiple hypothesis testing in gene and pathway analysis, we applied false discovery rate (FDR) correction using the Benjamini–Hochberg procedure. This ensures that significant results remain robust while minimizing false positives. For other scores, such as AUPRC, accuracy, F1, and recall, a bootstrapping statistical test with 1000 samplings is employed, and the significance of the difference in score medians is tested. The resulting *p* value is then corrected using the FDR method. In comparing the AUC of five‐fold crossvalidation between GPC‐NET and dense models across various sample sizes, a *t*‐test of the means is employed. The null hypothesis assumes that the two samples (GPC‐NET scores and dense scores) have identical average values, with the assumption that the populations have identical variances. The predictive performance of GPC‐Net was assessed using widely recognized machine learning metrics, including area under the receiver operating characteristic curve (AUC), area under the precision–recall curve (AUPRC), accuracy, F1‐score, precision, and recall (Figure 2d). These evaluation metrics are commonly used in bioinformatics and biomedical machine learning studies, particularly in disease classification and biomarker discovery [50, 51]. A nonparametric log‐rank test is utilized to compare estimates of the hazard functions of the two groups at each observed event time.

### 4.6 Selection of Important Neurons

To evaluate the relative importance of specific genes, pathways, and compounds contributing to the model prediction, we inspected the three hidden layers and used the DeepLIFT attribution method to obtain the total importance score of nodes.

DeepLIFT is a back propagation‐based attribution approach for assigning a sample‐level importance score for each feature. In this work, we are interested in assigning scores for each node in each layer. We used the “Rescale rule” of DeepLIFT to calculate the sample‐level importance of all nodes in all layers. Given a certain sample s, a specific target t and a node $x_{\mathrm{li}}$, denoting the ith node in layer l, the node importance score $C_{x_{\mathrm{li}}}$ can be calculated by averaged the sample‐level importance score over all testing samples, as follows:

$$C_{x_{\mathrm{li}}}=\frac{1}{N}|\sum_{s=1}^{N}C_{x_{\mathrm{li}}}^{s}|$$

where *N* is the number of testing samples, and $C_{x_{\mathrm{li}}}^{s}$ is the importance score of the node x_li_ over sample *s*.

The $C_{x_{\mathrm{li}}}^{s}$ is calculated over the following formula:

$$\Delta t=\sum_{i=1}^{\mathrm{nl}}C_{x_{\mathrm{li}}}^{s}$$

Where nl is the node number of layer l. Δt equals the sum of all node scores when fed by the given sample *S*, which denotes the difference in target activation *t*–*t*_0_, that is, $\mathrm{Dt}=t-t_{0}$.

### 4.7 The Protein Expression of ADCY8 in Breast Cancer Tissues

Normal tissues and cancer pathological tissue expression of ADCY8 proteins are from The Human Protein Atlas (https://www.proteinatlas.org/). The mean fluorescence density values were calculated by IMAGEJ software (https://imagej.nih.gov/ij/) while ensuring the same area of each fluorescence image. The mean fluorescence densities were compared using a two independent samples *t*‐test.

### 4.8 The mRNA and Protein Expression of ADCY8 in Breast Cancer Cell Lines

#### 4.8.1 Cell Culture

MCF‐7 and MDA‐MB‐231 cells were obtained from Shanghai Genechem (Shanghai, China) and maintained in Dulbecco's modified Eagle's medium (Invitrogen). SKBR3 cells were cultured in RPMI‐1640 medium (Invitrogen). All media were supplemented with 10% fetal bovine serum (FBS, HyClone). The human epithelial breast cell line, MCF‐10A, was maintained in DMEM containing 0.5 μg/mL hydrocortisone, 10 μg/mL insulin, 20 ng/mL human epidermal growth factor (EGF), and 5% heat‐inactivated horse serum.

#### 4.8.2 RNA Isolation and Quantitative Reverse Transcription Polymerase Chain Reaction (qRT‐PCR)

Total RNA was extracted using the Trizol reagent (SuperfecTRI, PufeiBio‐tech, China) and reverse transcribed to obtain cDNA, according to the manufacturer's protocol. Real‐time PCR was performed on LightCycler 480 I (Roche Diagnostics, Switzerland). Reagents were added to each tube as follows: SYBR premix ex taq 6.0 μL (DRR041B, TAKARA, Japan), Primer mix (5 μM) 0.3 μL (Genechem, China), template (reverse transcription product) 0.6 μL, RNase‐Free H_2_O 5.1 μL. The reference gene was ACTB (Upstream primer sequence: GCGTGACATTAAGGAGAAGC; downstream primer sequence: CCACGTCACACTTCATGATGG); the target gene was ADCY8 (Upstream primer sequence: CGGGATTTGGAACGCCTCT; downstream primer sequence: AGGTGACCACGCCGCTGTAC).

#### 4.8.3 Western Blot

Whole cell lysates were used for Western blotting analysis as previously described with the following modifications [52]: Cells were lysed using RIPA buffer (Beyotime) with a final concentration of 1 mM PMSF added immediately before use. The lysates were sonicated (40 W for 20 cycles, each cycle 1 s with a 2 s interval), and then centrifuged at 12,000 *g* for 15 min at 4°C. Protein concentration was determined using the BCA Protein Assay Kit (Beyotime), and equal amounts of protein (2 μg/μL) were separated on 10% SDS‐PAGE gels. Proteins were transferred to PVDF membranes (Millipore) using a wet transfer system at 200 mA for 120 min. Membranes were blocked with 5% nonfat milk in TBST for 1 h at room temperature, followed by incubation with anti‐ADCY8 antibody (ab196686, Abcam) or β‐actin monoclonal antibody (sc‐69879, Santa Cruz Biotechnology) at 4°C overnight. Secondary antibodies (antirabbit IgG: CST, #7074, antimouse IgG: CST, #7076) were incubated for 1.5 h at room temperature, and protein bands were visualized using the LumiGLO Reagent and Peroxide kit (CST, #7003).

### 4.9 Patients

Patient data were obtained from patients admitted to the Peking Union Medical College Hospital and were classified according to the histopathological findings of surgical resection as 51 breast invasive cancers and 28 noninvasive cancers (including carcinomas in situ or breast fibroadenomas). The study protocol was approved by the Medical Ethics Committee of PUMCH (approval number: K3993), and the requirement for obtaining written informed consent was waived. Samples that meet any of the following criteria are excluded: (a) Samples originating from duplicate collections by the same sample provider; (b) patients who do not meet the diagnostic criteria for breast tumors; (c) patients who have undergone surgical treatment, chemotherapy, or radiotherapy for breast cancer.

### 4.10 Detection of Urinary Tyrosine

Morning urine samples were collected from patients before surgery, stored in sterile urine cups, and then divided into two parts. The first part was used to determine urinary creatinine levels, while the second part, after adding a stabilizer (9:1 urine/stabilizer ratio), was stored at −20°C for later analysis of urinary tyrosine content. Urinary tyrosine was measured by liquid chromatography with tandem mass spectrometry system consisting of a Shimadzu Jasper HPLC chromatograph system (Shimadzu, Japan) and an AB SCIEX Triple QuadTM4500MD mass spectrometer (AB SCIEX, Foster City, CA, USA); chromatographic separation was performed using a XBridgeBEHAmidecolumn (2.1 × 100 mm; 2.5 μm). Multiple reaction monitoring (MRM) mode was used. Optimized parameters for mass detection were as follows: the MRM transitions were 182.1 → 136.1 for tyrosine and 188.1 → 142.1 for its internal standard (IS); the declustering potential was 50 eV and the collision energy was 40 eV (18 eV for IS); curtain gas was 20 kPa; ion spray voltage was 5500 V. The urinary tyrosine kit was purchased from haosibiotech. Urinary creatinine was measured by automatic analyzer (Roche C8000, Basel, Switzerland). We then corrected for amino acids with urine creatinine:

$$\frac{\mathrm{Tyr}\left(\text{μmol}/L\right)\times 1000\times 10/9}{\mathrm{Cr}\left(\text{mmol}/L\right)\times 113}$$

## 5 Conclusion

This study developed a novel sparse model, GPC‐Net, aimed at investigating breast cancer invasion and migration by integrating gene, pathway, and compound data. This Sparse neural network enables researchers to gain clearer insights into the genes and pathways involved in breast cancer progression and to directly observe the active compounds. This interpretable model surpasses traditional machine learning methods in prediction accuracy and provides new insights into the key genes and biological pathways associated with breast cancer.

## Author Contributions

**Xia Qian:** writing – original draft, validation, methodology, formal analysis, investigation, data curation, conceptualization, visualization. **Dandan Sun:** investigation, validation. **Yichen Ma:** validation, investigation. **Ling Qiu:** funding acquisition, supervision. **Jie Wu:** supervision, writing – review and editing.

## Ethics Statement

The all‐study protocol has been approved by the Medical Ethics Committee of PUMCH. For external validation, additional data were collected from inpatients at Peking Union Medical College Hospital, with the requirement for written informed consent waived by the Ethics Committee (approval number: K3993).

## Conflicts of Interest

The authors declare no conflicts of interest.

## Supporting information


Data S1.

## Data Availability

All the data are from public resources at https://www.cbioportal.org/. The main dataset was downloaded from (data, “Aparicio S et al.” cohort [46, 47, 48]). https://cbioportal‐datahub.s3.amazonaws.com/brca_metabric.tar.gz. The data of the external validation set were obtained from The Metastatic Breast Cancer Project (www.mbcproject.org), and the data can be downloaded from: https://cbioportal‐datahub.s3.amazonaws.com/brca_mbcproject_wagle_2017.tar.gz. We will permanently publish the developed code at https://github.com/286331722/Gene_Path_Compound_Neural_Network.
