# Supplementary material for: Exploring Mechanisms and Biomarkers of Breast Cancer Invasion and Migration: An Explainable Gene–Pathway–Compounds Neural Network
Source: Cancer Med. 2025 Mar 17;14(6):e70769. doi: 10.1002/cam4.70769 (PMC11912184; doi:10.1002/cam4.70769)
Supplement: Supplementary file 1 — Data S1. [file CAM4-14-e70769-s001.docx]

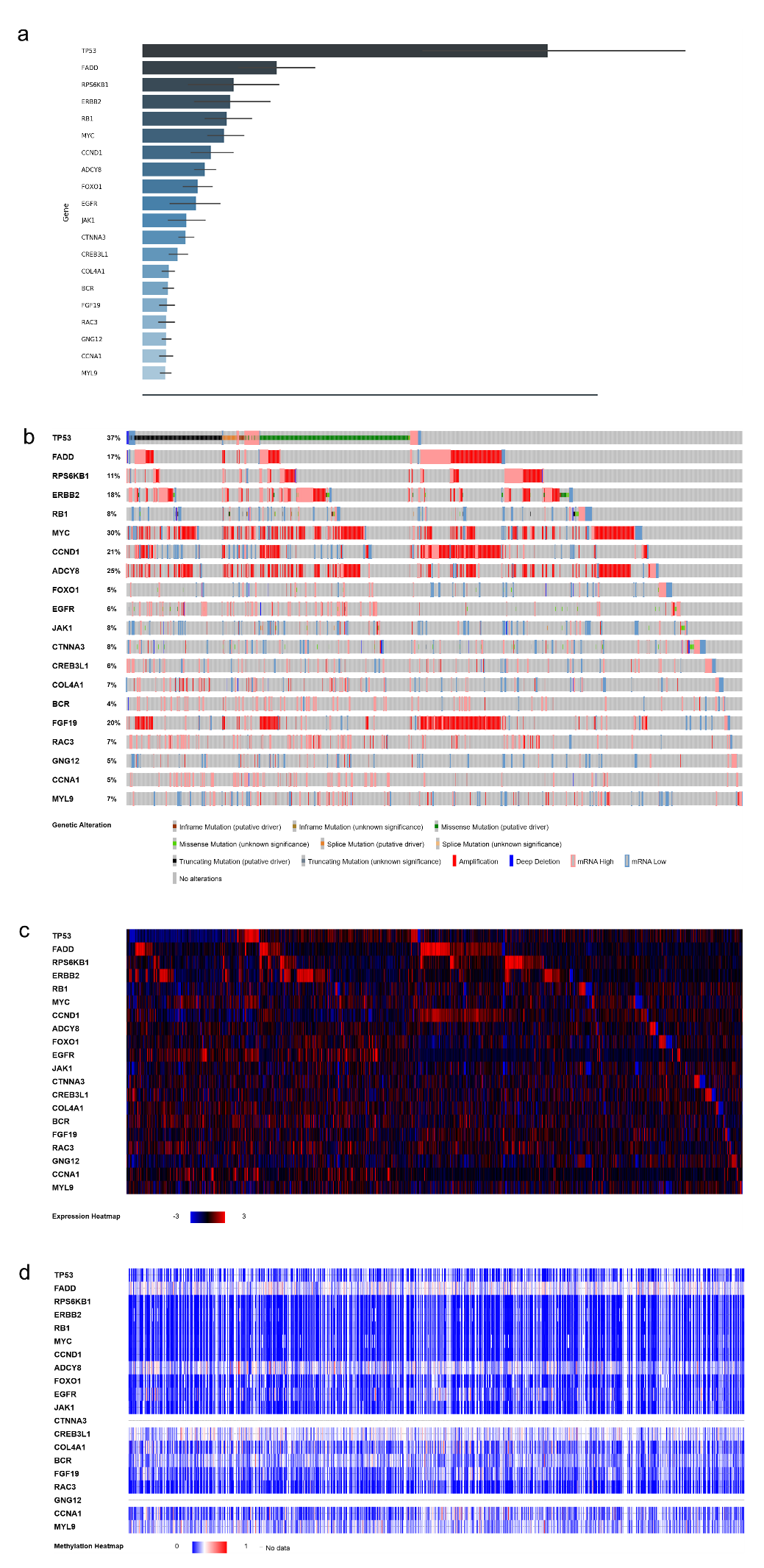


Extended Data Fig. 1 TOP gene.

a) The length of each gene bar was determined by adding the four input features, based on the relative ranking of the GPC-NET total significance scores. A random sample of 1000 samples from the test set was used to construct the confidence intervals using the ci method. b) Profile of molecular alterations in TOP genes. c), mRNA expression z-scores relative to all samples (log microarray). d), Promoter methylation (RRBS).


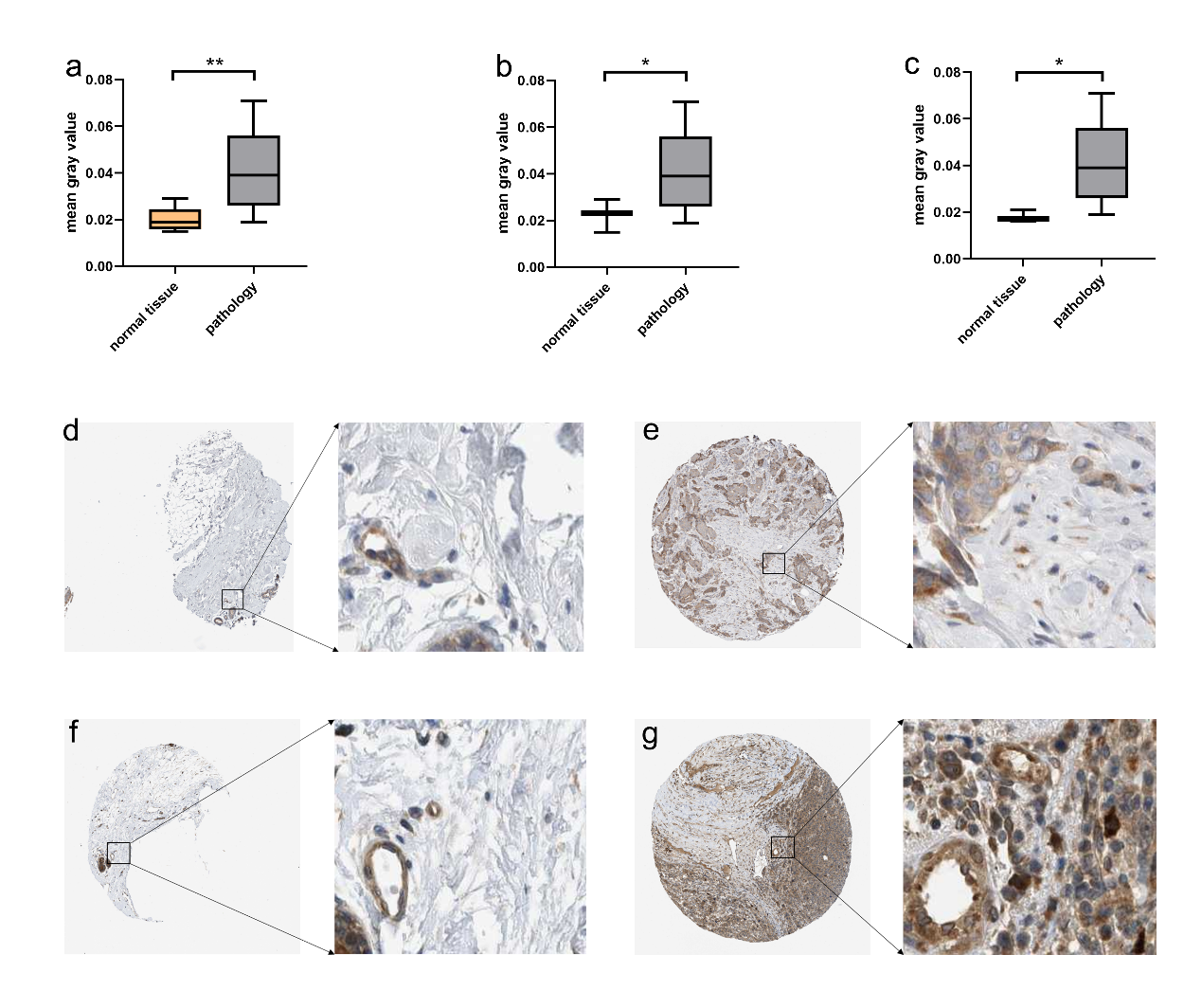


Extended Data Fig. 2 Comparison of ADCY8 protein expression in normal breast tissue and pathological breast cancer tissue

The mean gray values of anti-*ADCY8* antibody staining were compared between normal breast tissue and pathological breast cancer tissue. a)Staining results for all antibodies. b)Staining results for antibody HPA024291. c) Staining results for antibody CAB018652 (t test, *P < 0.05, **P < 0.01). d) Normal breast tissue stained with antibody HPA024291. e) Breast cancer pathology tissue stained with antibody HPA024291. f) Normal breast tissue stained with antibody CAB018652. g) Breast cancer pathology tissue stained with antibody CAB018652.


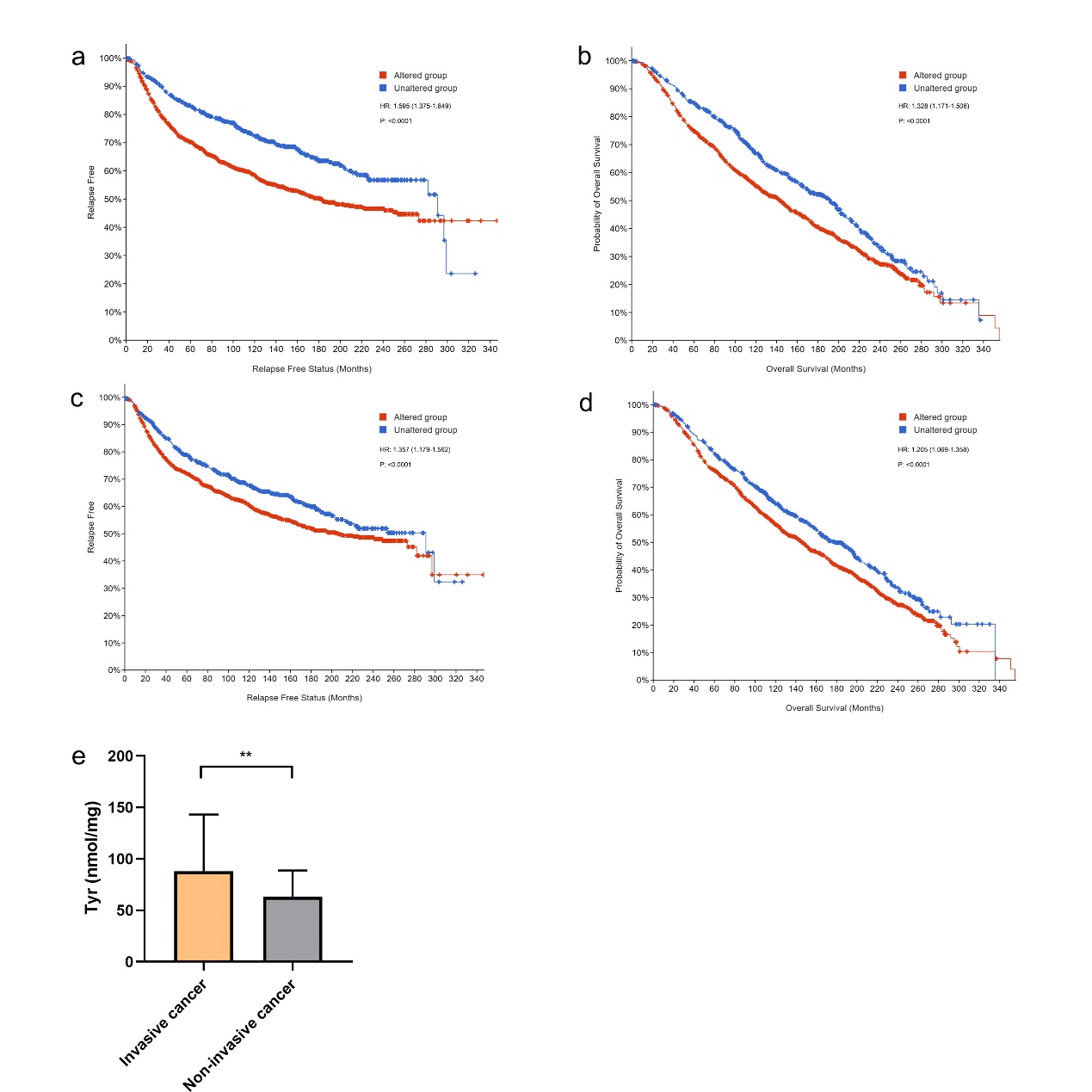


Extended Data Fig. 3 Analysis of the pathway layer

a) Relapse-free status curve of purine metabolism. Median months relapse free (95% CI): altered group, 175.03 (159.34 - 240.95); unaltered group, 290.89 (281.94 - NA), p<0.00001. b) Overall patient survival status curve of purine metabolism. Median months relapse free (95% CI): altered group, 143.17 (129.60 - 152.33); unaltered group, 191.93 (168.60 - 204.43), p<0.00001. c) Relapse free status curve of tyrosine metabolism. Median months relapse free (95% CI): altered group, 206.22 (170.69 - NA); unaltered group, 290.89 (213.19 - NA), p<0.001. d) Overall patient survival status curve of tyrosine metabolism. Median months relapse free (95% CI): altered group, 145.73 (132.20 - 155.37); unaltered group, 178.57 (160.33 - 197.73), p<0.00001. e) Urinary tyrosine comparison between patients with invasive and non-invasive breast cancer, **P < 0.01.

| 1. ADCY8 2. 140 kDa | **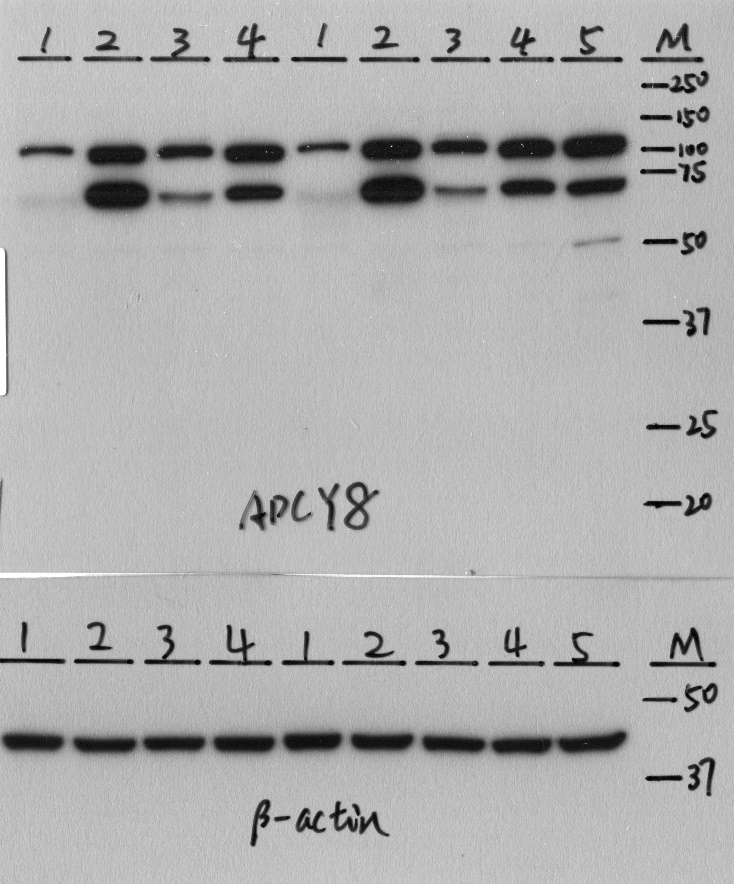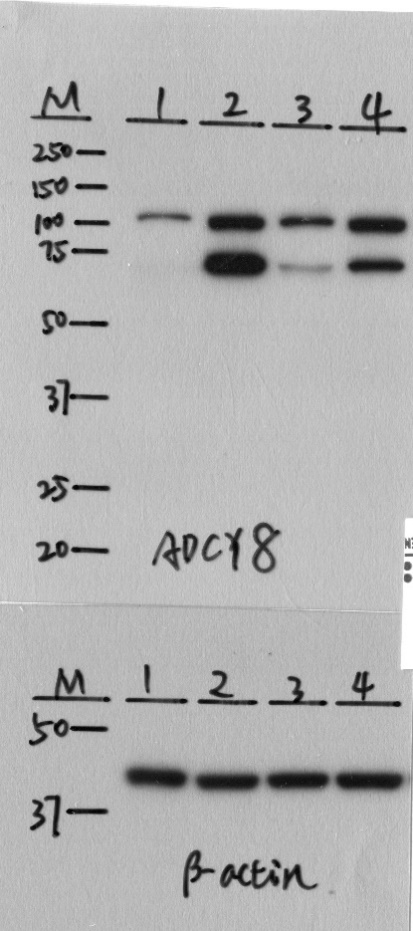** | | | | |
| --- | --- | --- | --- | --- | --- |
|  | 1. MCF 10A | 2. MCF7 | 3. SK-BR-3 | 4. MDA-MB-231 | 5. Hela |

Extended Data Fig. 4 Original Western blot (WB)

The target band detected in the target cells.1.MCF 10A. 2. MCF7. 3. SK-BR-3. 4. MDA-MB-231. 5. HeLa.

Extended table 1

Optimal hyperparameter

| hyper-parameters | optimum parameter |
| --- | --- |
| learning rate | 0.008 |
| batch size | 32 |
| optimizer | Adam |
| epoch | 50 |
| early stop | true |
| $\boldsymbol{\alpha}_{\mathbf{1}}$ | 2 |
| $\boldsymbol{\alpha}_{\mathbf{2}}$ | 20 |
| $\boldsymbol{\alpha}_{\mathbf{3}}$ | 400 |
| dropout | 0.5 |

Extended table 2

Metabolism pathway level

|  | KEGG id | Pathway Name | Coefficient of importance |
| --- | --- | --- | --- |
| Metabolism pathway | hsa00230 | Purine metabolism | 0.730156 |
|  | hsa00140 | Steroid hormone biosynthesis | 0.08663 |
|  | hsa00564 | Glycerophospholipid metabolism | 0.055122 |
|  | hsa00600 | Sphingolipid metabolism | 0.04284 |
|  | hsa00510 | N-Glycan biosynthesis | 0.042532 |
|  | hsa00590 | Arachidonic acid metabolism | 0.03327 |
|  | hsa00520 | Amino sugar and nucleotide sugar metabolism | 0.031321 |
|  | hsa00010 | Glycolysis / Gluconeogenesis | 0.02243 |
|  | hsa00982 | Drug metabolism - cytochrome P450 | 0.021608 |
|  | hsa00270 | Cysteine and methionine metabolism | 0.020967 |
|  | hsa00480 | Glutathione metabolism | 0.02082 |
|  | hsa00380 | Tryptophan metabolism | 0.015445 |
|  | hsa00760 | Nicotinate and nicotinamide metabolism | 0.014521 |
|  | hsa00790 | Folate biosynthesis | 0.011975 |
|  | hsa00020 | Citrate cycle (TCA cycle) | 0.007874 |
|  | hsa00190 | Oxidative phosphorylation | 0.007107 |
|  | hsa00310 | Lysine degradation | 0.007087 |
|  | hsa00640 | Propanoate metabolism | 0.006882 |
|  | hsa00630 | Glyoxylate and dicarboxylate metabolism | 0.00667 |
|  | hsa00860 | Porphyrin metabolism | 0.006131 |
|  | hsa00500 | Starch and sucrose metabolism | 0.005181 |
|  | hsa00830 | Retinol metabolism | 0.00514 |
|  | hsa00900 | Terpenoid backbone biosynthesis | 0.004702 |
|  | hsa00591 | Linoleic acid metabolism | 0.004491 |
|  | hsa00601 | Glycosphingolipid biosynthesis - lacto a | 0.004196 |
|  | hsa00440 | Phosphonate and phosphinate metabolism | 0.003287 |
|  | hsa00650 | Butanoate metabolism | 0.003091 |
|  | hsa00562 | Inositol phosphate metabolism | 0.003069 |
|  | hsa00770 | Pantothenate and CoA biosynthesis | 0.003002 |
|  | hsa00040 | Pentose and glucuronate interconversions | 0.00275 |
|  | hsa00350 | Tyrosine metabolism | 0.002624 |
|  | hsa00565 | Ether lipid metabolism | 0.002182 |
|  | hsa00410 | beta-Alanine metabolism | 0.001771 |
|  | hsa00561 | Glycerolipid metabolism | 0.001727 |
|  | hsa00030 | Pentose phosphate pathway | 0.00148 |
|  | hsa00983 | Drug metabolism - other enzymes | 0.001327 |
|  | hsa00052 | Galactose metabolism | 0.001285 |
|  | hsa00980 | Metabolism of xenobiotics by cytochrome | 0.001222 |
|  | hsa00340 | Histidine metabolism | 0.001132 |
|  | hsa00563 | Glycosylphosphatidylinositol (GPI)-ancho | 0.001104 |
|  | hsa01040 | Biosynthesis of unsaturated fatty acids | 0.000975 |
|  | hsa00330 | Arginine and proline metabolism | 0.000936 |
|  | hsa00513 | Various types of N-glycan biosynthesis | 0.000897 |
|  | hsa00100 | Steroid biosynthesis | 0.000807 |
|  | hsa00071 | Fatty acid degradation | 0.00075 |
|  | hsa00360 | Phenylalanine metabolism | 0.00065 |
|  | hsa00062 | Fatty acid elongation | 0.000645 |
|  | hsa00620 | Pyruvate metabolism | 0.000629 |
|  | hsa00240 | Pyrimidine metabolism | 0.000556 |
|  | hsa00120 | Primary bile acid biosynthesis | 0.00055 |
|  | hsa00430 | Taurine and hypotaurine metabolism | 0.000538 |
|  | hsa00051 | Fructose and mannose metabolism | 0.000524 |
|  | hsa00910 | Nitrogen metabolism | 0.000509 |
|  | hsa00061 | Fatty acid biosynthesis | 0.000463 |
|  | hsa00604 | Glycosphingolipid biosynthesis - ganglio | 0.00045 |
|  | hsa00470 | D-Amino acid metabolism | 0.000418 |
|  | hsa00592 | alpha-Linolenic acid metabolism | 0.000402 |
|  | hsa00603 | Glycosphingolipid biosynthesis - globo a | 0.000391 |
|  | hsa00450 | Selenocompound metabolism | 0.000359 |
|  | hsa00730 | Thiamine metabolism | 0.000238 |
|  | hsa00232 | Caffeine metabolism | 0.000123 |
|  | hsa00130 | Ubiquinone and other terpenoid-quinone b | 0.000117 |
|  | hsa00670 | One carbon pool by folate | 0.000105 |
|  | hsa00534 | Glycosaminoglycan biosynthesis - heparan | 9.85E-05 |
|  | hsa00785 | Lipoic acid metabolism | 8.60E-05 |
|  | hsa00515 | Mannose type O-glycan biosynthesis | 7.57E-05 |
|  | hsa00512 | Mucin type O-glycan biosynthesis | 6.81E-05 |
|  | hsa00220 | Arginine biosynthesis | 6.28E-05 |
|  | hsa00920 | Sulfur metabolism | 6.23E-05 |
|  | hsa00053 | Ascorbate and aldarate metabolism | 5.65E-05 |
|  | hsa00750 | Vitamin B6 metabolism | 5.02E-05 |
|  | hsa00531 | Glycosaminoglycan degradation | 3.31E-05 |
|  | hsa00740 | Riboflavin metabolism | 3.05E-05 |
|  | hsa00532 | Glycosaminoglycan biosynthesis - chondro | 2.75E-05 |
|  | hsa00780 | Biotin metabolism | 9.16E-07 |
|  | hsa01100 | Metabolic pathways | 0 |
|  | hsa01200 | Carbon metabolism | 0 |
|  | hsa01210 | 2-Oxocarboxylic acid metabolism | 0 |
|  | hsa01212 | Fatty acid metabolism | 0 |
|  | hsa01230 | Biosynthesis of amino acids | 0 |
|  | hsa01232 | Nucleotide metabolism | 0 |
|  | hsa01250 | Biosynthesis of nucleotide sugars | 0 |
|  | hsa01240 | Biosynthesis of cofactors | 0 |
|  | hsa00250 | Alanine, aspartate and glutamate metabol | 0 |
|  | hsa00260 | Glycine, serine and threonine metabolism | 0 |
|  | hsa00280 | Valine, leucine and isoleucine degradati | 0 |
|  | hsa00290 | Valine, leucine and isoleucine biosynthe | 0 |
|  | hsa00400 | Phenylalanine, tyrosine and tryptophan b | 0 |
|  | hsa00524 | Neomycin, kanamycin and gentamicin biosy | 0 |
|  | hsa00533 | Glycosaminoglycan biosynthesis - keratan | 0 |
|  | hsa00511 | Other glycan degradation | 0 |
|  | hsa00514 | Other types of O-glycan biosynthesis | 0 |
| Amino acid metabolism | hsa00270 | Cysteine and methionine metabolism | 0.020967 |
|  | hsa00480 | Glutathione metabolism | 0.02082 |
|  | hsa00380 | Tryptophan metabolism | 0.015445 |
|  | hsa00310 | Lysine degradation | 0.007087 |
|  | hsa00350 | Tyrosine metabolism | 0.002624 |
|  | hsa00410 | beta-Alanine metabolism | 0.001771 |
|  | hsa00340 | Histidine metabolism | 0.001132 |
|  | hsa00330 | Arginine and proline metabolism | 0.000936 |
|  | hsa00360 | Phenylalanine metabolism | 0.00065 |
|  | hsa00430 | Taurine and hypotaurine metabolism | 0.000538 |
|  | hsa00470 | D-Amino acid metabolism | 0.000418 |
|  | hsa00220 | Arginine biosynthesis | 6.28E-05 |
|  | hsa00250 | Alanine, aspartate and glutamate metabol | 0 |
|  | hsa00400 | Phenylalanine, tyrosine and tryptophan b | 0 |
|  | hsa00290 | Valine, leucine and isoleucine biosynthe | 0 |
|  | hsa00280 | Valine, leucine and isoleucine degradati | 0 |
|  | hsa00260 | Glycine, serine and threonine metabolism | 0 |
| Carbohydrate metabolism | hsa00520 | Amino sugar and nucleotide sugar metabol | 0.031321 |
|  | hsa00010 | Glycolysis / Gluconeogenesis | 0.02243 |
|  | hsa00020 | Citrate cycle (TCA cycle) | 0.007874 |
|  | hsa00640 | Propanoate metabolism | 0.006882 |
|  | hsa00630 | Glyoxylate and dicarboxylate metabolism | 0.00667 |
|  | hsa00500 | Starch and sucrose metabolism | 0.005181 |
|  | hsa00650 | Butanoate metabolism | 0.003091 |
|  | hsa00562 | Inositol phosphate metabolism | 0.003069 |
|  | hsa00040 | Pentose and glucuronate interconversions | 0.00275 |
|  | hsa00030 | Pentose phosphate pathway | 0.00148 |
|  | hsa00052 | Galactose metabolism | 0.001285 |
|  | hsa00620 | Pyruvate metabolism | 0.000629 |
|  | hsa00051 | Fructose and mannose metabolism | 0.000524 |
|  | hsa00053 | Ascorbate and aldarate metabolism | 5.65E-05 |
| Energy metabolism | hsa00270 | Cysteine and methionine metabolism | 0.020967 |
|  | hsa00480 | Glutathione metabolism | 0.02082 |
|  | hsa00380 | Tryptophan metabolism | 0.015445 |
|  | hsa00310 | Lysine degradation | 0.007087 |
|  | hsa00350 | Tyrosine metabolism | 0.002624 |
|  | hsa00410 | beta-Alanine metabolism | 0.001771 |
|  | hsa00340 | Histidine metabolism | 0.001132 |
|  | hsa00330 | Arginine and proline metabolism | 0.000936 |
|  | hsa00360 | Phenylalanine metabolism | 0.000649 |
|  | hsa00430 | Taurine and hypotaurine metabolism | 0.000538 |
|  | hsa00470 | D-Amino acid metabolism | 0.000418 |
|  | hsa00220 | Arginine biosynthesis | 6.28E-05 |
|  | hsa00250 | Alanine, aspartate and glutamate metabol | 0 |
|  | hsa00260 | Glycine, serine and threonine metabolism | 0 |
|  | hsa00280 | Valine, leucine and isoleucine degradati | 0 |
|  | hsa00290 | Valine, leucine and isoleucine biosynthe | 0 |
|  | hsa00400 | Phenylalanine, tyrosine and tryptophan b | 0 |
| Genetic Information Processing | hsa04141 | Protein processing in endoplasmic reticulum | 0.121776 |
|  | hsa00970 | Aminoacyl-tRNA biosynthesis | 0.009408 |
|  | hsa04122 | Sulfur relay system | 0.000203 |
|  | hsa03020 | RNA polymerase | 0 |
|  | hsa03022 | Basal transcription factors | 0 |
|  | hsa03040 | Spliceosome | 0 |
|  | hsa03010 | Ribosome | 0 |
|  | hsa03013 | Nucleocytoplasmic transport | 0 |
|  | hsa03015 | mRNA surveillance pathway | 0 |
|  | hsa03008 | Ribosome biogenesis in eukaryotes | 0 |
|  | hsa03060 | Protein export | 0 |
|  | hsa04130 | SNARE interactions in vesicular transport | 0 |
|  | hsa04120 | Ubiquitin mediated proteolysis | 0 |
|  | hsa03050 | Proteasome | 0 |
|  | hsa03018 | RNA degradation | 0 |
|  | hsa03030 | DNA replication | 0 |
|  | hsa03410 | Base excision repair | 0 |
|  | hsa03420 | Nucleotide excision repair | 0 |
|  | hsa03430 | Mismatch repair | 0 |
|  | hsa03440 | Homologous recombination | 0 |
|  | hsa03450 | Non-homologous end-joining | 0 |
|  | hsa03460 | Fanconi anemia pathway | 0 |
| Lipid metabolism | hsa00140 | Steroid hormone biosynthesis | 0.08663 |
|  | hsa00564 | Glycerophospholipid metabolism | 0.055122 |
|  | hsa00600 | Sphingolipid metabolism | 0.04284 |
|  | hsa00590 | Arachidonic acid metabolism | 0.03327 |
|  | hsa00591 | Linoleic acid metabolism | 0.004491 |
|  | hsa00565 | Ether lipid metabolism | 0.002182 |
|  | hsa00561 | Glycerolipid metabolism | 0.001727 |
|  | hsa01040 | Biosynthesis of unsaturated fatty acids | 0.000975 |
|  | hsa00100 | Steroid biosynthesis | 0.000807 |
|  | hsa00071 | Fatty acid degradation | 0.00075 |
|  | hsa00062 | Fatty acid elongation | 0.000645 |
|  | hsa00120 | Primary bile acid biosynthesis | 0.00055 |
|  | hsa00061 | Fatty acid biosynthesis | 0.000463 |
|  | hsa00592 | alpha-Linolenic acid metabolism | 0.000402 |
| Organismal Systems | hsa04211 | Longevity regulating pathway | 3.055276 |
|  | hsa04261 | Adrenergic signaling in cardiomyocytes | 0.637073 |
|  | hsa04666 | Fc gamma R-mediated phagocytosis | 0.590968 |
|  | hsa04213 | Longevity regulating pathway - multiple species | 0.588722 |
|  | hsa04974 | Protein digestion and absorption | 0.488109 |
|  | hsa04910 | Insulin signaling pathway | 0.397526 |
|  | hsa04714 | Thermogenesis | 0.355187 |
|  | hsa04924 | Renin secretion | 0.30071 |
|  | hsa04062 | Chemokine signaling pathway | 0.297689 |
|  | hsa04658 | Th1 and Th2 cell differentiation | 0.211315 |
|  | hsa04923 | Regulation of lipolysis in adipocytes | 0.167944 |
|  | hsa04916 | Melanogenesis | 0.117352 |
|  | hsa04721 | Synaptic vesicle cycle | 0.075427 |
|  | hsa04919 | Thyroid hormone signaling pathway | 0.064699 |
|  | hsa04722 | Neurotrophin signaling pathway | 0.039616 |
|  | hsa04662 | B cell receptor signaling pathway | 0.020531 |
|  | hsa04720 | Long-term potentiation | -0.0081 |
|  | hsa04660 | T cell receptor signaling pathway | -0.02331 |
|  | hsa04625 | C-type lectin receptor signaling pathway | -0.11499 |
|  | hsa04726 | Serotonergic synapse | -0.12311 |
|  | hsa04610 | Complement and coagulation cascades | -0.1596 |
|  | hsa04723 | Retrograde endocannabinoid signaling | -0.1608 |
|  | hsa04725 | Cholinergic synapse | -0.16455 |
|  | hsa04973 | Carbohydrate digestion and absorption | -0.17478 |
|  | hsa04978 | Mineral absorption | -0.17823 |
|  | hsa04970 | Salivary secretion | -0.19656 |
|  | hsa04913 | Ovarian steroidogenesis | -0.20591 |
|  | hsa04979 | Cholesterol metabolism | -0.22061 |
|  | hsa04960 | Aldosterone-regulated sodium reabsorption | -0.22366 |
|  | hsa04921 | Oxytocin signaling pathway | -0.23504 |
|  | hsa04621 | NOD-like receptor signaling pathway | -0.2404 |
|  | hsa04380 | Osteoclast differentiation | -0.24769 |
|  | hsa04360 | Axon guidance | -0.26445 |
|  | hsa03320 | PPAR signaling pathway | -0.27185 |
|  | hsa04972 | Pancreatic secretion | -0.28368 |
|  | hsa04961 | Endocrine and other factor-regulated calcium reabsorption | -0.29814 |
|  | hsa04727 | GABAergic synapse | -0.30467 |
|  | hsa04670 | Leukocyte transendothelial migration | -0.30483 |
|  | hsa04964 | Proximal tubule bicarbonate reclamation | -0.30907 |
|  | hsa04912 | GnRH signaling pathway | -0.31193 |
|  | hsa04728 | Dopaminergic synapse | -0.31433 |
|  | hsa04724 | Glutamatergic synapse | -0.31742 |
|  | hsa04650 | Natural killer cell mediated cytotoxicity | -0.32461 |
|  | hsa04915 | Estrogen signaling pathway | -0.32774 |
|  | hsa04613 | Neutrophil extracellular trap formation | -0.32802 |
|  | hsa04270 | Vascular smooth muscle contraction | -0.33118 |
|  | hsa04664 | Fc epsilon RI signaling pathway | -0.33581 |
|  | hsa04922 | Glucagon signaling pathway | -0.33861 |
|  | hsa04926 | Relaxin signaling pathway | -0.33995 |
|  | hsa04920 | Adipocytokine signaling pathway | -0.34402 |
|  | hsa04742 | Taste transduction | -0.3447 |
|  | hsa04710 | Circadian rhythm | -0.34512 |
|  | hsa04914 | Progesterone-mediated oocyte maturation | -0.3458 |
|  | hsa04918 | Thyroid hormone synthesis | -0.34582 |
|  | hsa04713 | Circadian entrainment | -0.34991 |
|  | hsa04750 | Inflammatory mediator regulation of TRP channels | -0.35403 |
|  | hsa04976 | Bile secretion | -0.35795 |
|  | hsa04659 | Th17 cell differentiation | -0.35907 |
|  | hsa04611 | Platelet activation | -0.35943 |
|  | hsa04929 | GnRH secretion | -0.36016 |
|  | hsa04917 | Prolactin signaling pathway | -0.361 |
|  | hsa04911 | Insulin secretion | -0.36148 |
|  | hsa04260 | Cardiac muscle contraction | -0.3629 |
|  | hsa04925 | Aldosterone synthesis and secretion | -0.36444 |
|  | hsa04971 | Gastric acid secretion | -0.36546 |
|  | hsa04975 | Fat digestion and absorption | -0.36598 |
|  | hsa04730 | Long-term depression | -0.36624 |
|  | hsa04623 | Cytosolic DNA-sensing pathway | -0.36631 |
|  | hsa04966 | Collecting duct acid secretion | -0.36848 |
|  | hsa04744 | Phototransduction | -0.36993 |
|  | hsa04672 | Intestinal immune network for IgA production | -0.37148 |
|  | hsa04740 | Olfactory transduction | -0.37226 |
|  | hsa04962 | Vasopressin-regulated water reabsorption | -0.37233 |
|  | hsa04614 | Renin-angiotensin system | -0.37266 |
|  | hsa04977 | Vitamin digestion and absorption | -0.37311 |
|  | hsa04927 | Cortisol synthesis and secretion | -0.37335 |
|  | hsa04622 | RIG-I-like receptor signaling pathway | -0.37344 |
|  | hsa04640 | Hematopoietic cell lineage | -0.37361 |
|  | hsa04620 | Toll-like receptor signaling pathway | -0.37361 |
|  | hsa04612 | Antigen processing and presentation | -0.37361 |
|  | hsa04657 | IL-17 signaling pathway | -0.37361 |
|  | hsa04935 | Growth hormone synthesis, secretion and action | -0.37361 |
|  | hsa04928 | Parathyroid hormone synthesis, secretion and action | -0.37361 |
